# Severe right ventricular infarction due to iatrogenic aortocoronary dissection successfully treated by surgical repair and extracorporeal membrane oxygenation

**DOI:** 10.1093/jscr/rjad711

**Published:** 2024-01-03

**Authors:** Shunya Ono, Shuhei Kawamoto, Toshiya Fukushima, Motoharu Shimozawa, Retsu Tateishi, Fumiya Haba, Yoshinori Nakahara, Takeyuki Kanemura

**Affiliations:** Department of Cardiovascular Surgery, IMS Katsushika Heart Center, 3-30-1 Horikiri, Katsushika, Tokyo 124-0006, Japan; Department of Cardiovascular Surgery, IMS Katsushika Heart Center, 3-30-1 Horikiri, Katsushika, Tokyo 124-0006, Japan; Department of Cardiovascular Surgery, IMS Katsushika Heart Center, 3-30-1 Horikiri, Katsushika, Tokyo 124-0006, Japan; Department of Cardiovascular Surgery, IMS Katsushika Heart Center, 3-30-1 Horikiri, Katsushika, Tokyo 124-0006, Japan; Department of Cardiovascular Surgery, IMS Katsushika Heart Center, 3-30-1 Horikiri, Katsushika, Tokyo 124-0006, Japan; Department of Cardiovascular Surgery, IMS Katsushika Heart Center, 3-30-1 Horikiri, Katsushika, Tokyo 124-0006, Japan; Department of Cardiovascular Surgery, IMS Katsushika Heart Center, 3-30-1 Horikiri, Katsushika, Tokyo 124-0006, Japan; Department of Cardiovascular Surgery, IMS Katsushika Heart Center, 3-30-1 Horikiri, Katsushika, Tokyo 124-0006, Japan

**Keywords:** iatrogenic aortocoronary dissection, right ventricular infarction, extracorporeal membrane oxygenation

## Abstract

Iatrogenic aortocoronary dissection (IACD) is a rare but potentially fatal complication of percutaneous coronary intervention or coronary angiography (CAG). In particular, if the condition of the patient is complicated by cardiogenic shock and right ventricular (RV) dysfunction, the mortality rate is high. Herein, we report the case of an 85-year-old woman with IACD who underwent elective CAG of the right coronary artery complicated with cardiogenic shock due to RV infarction. After prompt surgical repair and postoperative extracorporeal membrane oxygenation, the postoperative course was uneventful and the patient was discharged to a rehabilitation facility.

## Introduction

Iatrogenic aortocoronary dissection (IACD) during percutaneous coronary intervention (PCI) or coronary angiography (CAG) is an extremely rare but potentially lethal complication, occurring in <0.1% of procedures [1]. The early mortality rate of IACD requiring surgical intervention is reported to be quite high, especially in patients with IACD that occurred after diagnostic CAG [2]. Furthermore, the prognosis of patients with right ventricular infarction and cardiogenic shock is extremely poor according to previous studies [3].

Herein, we report a rare case of a woman with IACD accompanied by severe right ventricular (RV) infarction during CAG, successfully treated with surgical repair and extracorporeal membrane oxygenation (ECMO).

## Case presentation

**Figure 1:**
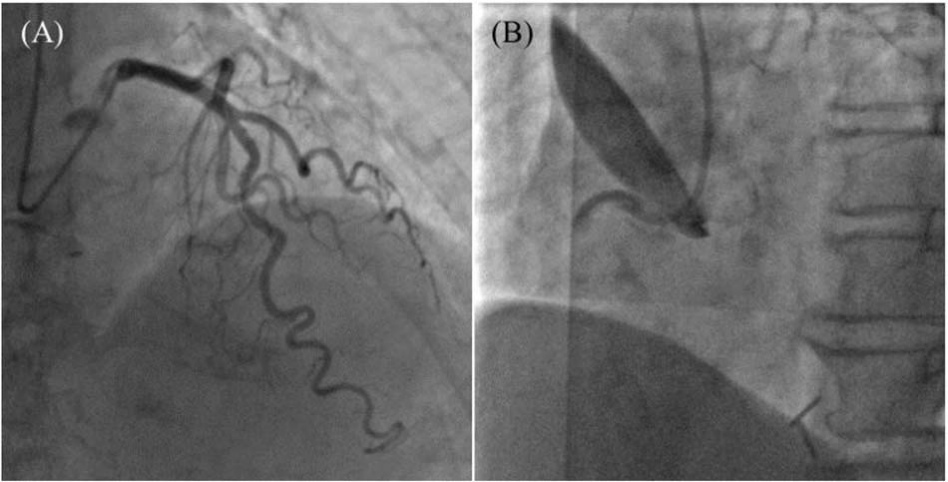
Coronary angiography images. (A) Intact left coronary artery without collateral blood supply into the right coronary artery (RCA). (B) Aortocoronary dissection of RCA with total occlusion.

**Figure 2:**
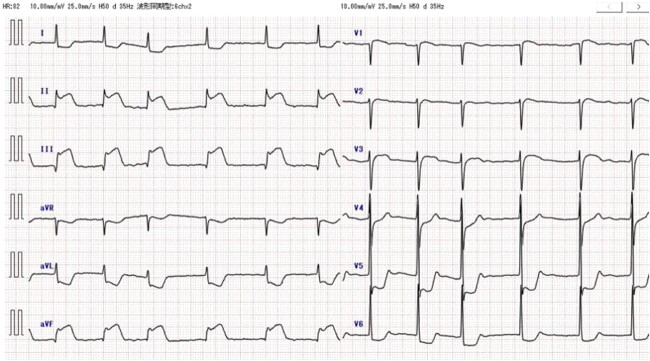
Preoperative electrocardiography. ST elevation in leads II, III, and aVF, and reciprocal ST depression in leads I, aVL, and V4-6 were detected.

**Figure 3:**
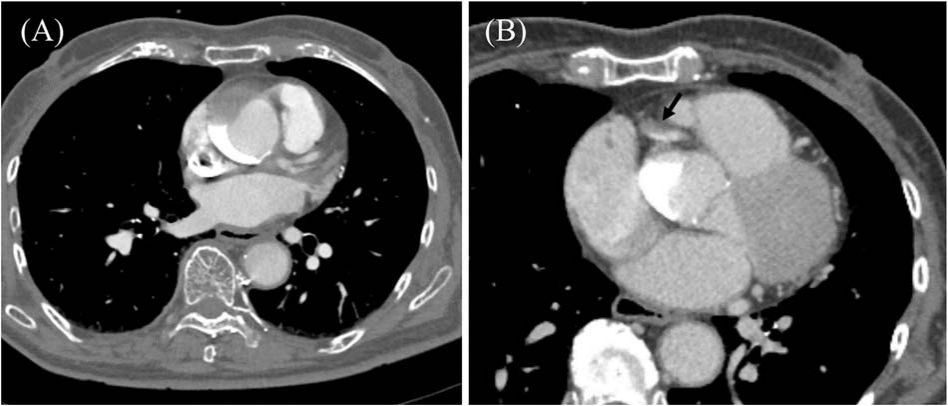
Preoperative contrast-enhanced CT. (A) Ascending aortic dissection is detected. Contrast-material is pooled in the false lumen. (B) Dissection at the proximal part of RCA is suspected (black arrow).

An 85-year-old woman with a history of hypertension and cerebral infarction presented to the emergency department of another hospital with the complaint of temporary loss of consciousness (LOC). Dehydration was suspected to be the cause of LOC; however, CAG was performed to confirm the reason for the mild elevation in creatine kinase and D-dimer levels. While the left coronary system was intact (Fig. 1A), the coronary dissection spread retrogradely from the right sinus of Valsalva to the ascending aorta after injecting contrast into the right coronary artery (RCA). The RCA was completely occluded at the proximal part, and no collateral flow into the RCA was observed (Fig. 1B). Electrocardiography revealed previously undetected ST elevation in leads II, III, and aVF and reciprocal ST depression in leads I, aVL, and V4–6 (Fig. 2). Contrast-enhanced CT confirmed aortic dissection which was localized in the ascending aorta (Fig. 3A). Additionally, RCA occlusion at the proximal portion was suspected (Fig. 3B). The patient was transferred to our institute for further management of the aortocoronary dissection. On arrival, the patient was alert and oriented with close to normal vital signs. Echocardiography showed a normal left ventricular ejection fraction with preserved RV function; however, severe hypokinesis of the inferior wall was noted. Moreover, as the hemodynamic status of the patient gradually deteriorated in the emergency department, we decided to perform an emergency surgery.

In the operating theater, we performed transesophageal echocardiography, which confirmed that the dissection had extended to the descending aorta. Cardiopulmonary bypass (CPB) was initiated with an arterial cannula inserted into the left femoral artery and a venous cannula via the right atrium. Cardiac arrest was induced by clamping the ascending aorta and inducing retrograde cardioplegia through the coronary sinuses. Subsequently, moderate hypothermia (bladder temperature, 28°C) was induced, and the ascending aorta was opened. Only retrograde cerebral perfusion was used for cerebral protection during circulatory arrest. Intimal tears were not visible in the ascending aorta, and the false lumen was partially thrombosed. Ascending aortic replacement with a single-branched dacron graft (Triplex® 26/10 mm; Terumo Corporation, Tokyo, Japan) under circulatory arrest was performed. This was followed by a single-vessel coronary artery bypass graft in the middle section of the RCA, using a saphenous vein graft and ligation of the ostium of RCA. Though the motion of the left ventricular inferior wall improved significantly, weaning from CPB was unsuccessful due to severe RV dysfunction that did not improve with high-dose catecholamines and nitric oxide inhalation. Subsequently, peripheral venoarterial ECMO was initiated.

Post-operative echocardiography revealed the gradual recovery of RV function. The patient was weaned off ECMO on postoperative day (POD) 5 and extubated on POD 9. Post-operative contrast-enhanced CT revealed no residual dissection, and the bypass graft was patent. The post-operative course was uncomplicated, and the patient was transferred to a rehabilitation facility on POD 18.

## Discussion

IACD during PCI or CAG is an extremely rare, but nonetheless catastrophic complication, occurring in <0.1% of procedures [1]. The underlying cause of IACD in the present case is unclear; however, this may be attributed to the forceful manipulation of the guiding catheter or guidewire and blind injection of contrast material [4].

The prognosis of patients with IACD who were managed with percutaneous coronary intervention (PCI) or conservatively was excellent; however, patients who required surgical intervention had an extremely poor prognosis [2, 5]. Biancari *et al*. [2] analyzed the causes of short- and long-term mortality in patients with iatrogenic aortic dissections. They concluded that the in-hospital mortality in patients with IACD after diagnostic CAG was >40%, which was comparatively worse than that in patients with spontaneous Stanford type A aortic dissection and IACD after cardiac surgery and PCI.

The present patient had an inferior myocardial infarction complicated by severe RV dysfunction, which resulted in rapid hemodynamic deterioration in the emergency room. Moreover, patients with inferior myocardial infarction and RV dysfunction may have a worse prognosis than those without RV involvement. Additionally, extremely high in-hospital mortality in patients with predominant cardiogenic shock due to RV infarction has been reported [3]. The cause of severe right ventricular infarction may be attributed to the absence of preoperative coronary artery lesions on the RCA, as well as the lack of a well-developed collateral blood supply to the RCA. Therefore, sudden proximal occlusion of the RCA may lead to massive right ventricular infarction [6]. In addition to surgical revascularization, medical management with catecholamines and inhaled nitric oxide, which is considered effective for patients with cardiogenic shock due to RV dysfunction [7], was attempted; however, it did not show sufficient improvement. The initiation of venoarterial ECMO is necessary because of the challenges in weaning off CPB. Owing to the preserved left ventricular function in this patient, a decrease in right atrial and ventricular preload was obtained without increasing the left atrial and pulmonary arterial pressure, which resulted in the recovery of RV function.

In conclusion, IACD accompanied by RV infarction is a devastating complication with high mortality risk. If a patient is hemodynamically unstable, prompt surgical intervention and proper post-operative management are mandatory, which may further lead to positive outcomes.
